# Using network pharmacology and molecular docking technology, proteomics and experiments were used to verify the effect of Yigu decoction (YGD) on the expression of key genes in osteoporotic mice

**DOI:** 10.1080/07853890.2024.2449225

**Published:** 2025-01-03

**Authors:** Kun Yan, Rui-Kun Zhang, Jia-Xin Wang, Hai-Feng Chen, Yang Zhang, Feng Cheng, Yi Jiang, Min Wang, Ziqi Wu, Xiao-Gang Chen, Zhi-Neng Chen, Gui-Jin Li, Xin-Miao Yao

**Affiliations:** a The Third Affiliated Hospital of Zhejiang Chinese Medical University, Hangzhou, China; b The Third Clinical Medical College of Zhejiang Chinese Medical University, Hangzhou, China

**Keywords:** Osteoporosis, Yigu decoction, network pharmacology, molecular docking, bioinformatics, molecular mechanism

## Abstract

**Background:**

Yigu decoction (YGD) is a traditional Chinese medicine prescription for the treatment of osteoporosis, although many clinical studies have confirmed its anti-OP effect, but the specific mechanism is still not completely clear.

**Methods:**

In this study, through the methods of network pharmacology and molecular docking, the material basis and action target of YGD in preventing and treating OP were analyzed, and the potential target and mechanism of YGD in preventing and treating OP were clarified by TMT quantitative protein and experiment.

**Results:**

Network pharmacology and molecular docking revealed that the active components of YGD were mainly stigmasterol and flavonoids. Molecular docking mainly studied the strong binding ability of stigmasterol to the target. Animal proteomics verified the related mechanism of YGD in preventing and treating OP. Based on the KEGG enrichment of network pharmacology and histology, our animal experiments *in vivo* verified that YGD may play a role in the treatment of OP by mediating hif1- α/vegf/glut1 signal pathway.

**Conclusions:**

YGD prevention and treatment of OP may be achieved by interfering with multiple targets. This study confirmed that it may promote osteoblast proliferation and protect osteoblast function by up-regulating the expression of proteins related to HIF signal pathway.

## 1. Introduction

OP is a metabolic disease characterized by low bone mass, destruction of bone tissue structure and increase of bone fragility, which increase of age, the probability of the disease increases [1,2]. Because the onset of OP is hidden [3], most patients with OP can only be diagnosed during physical examination or even fracture [4,5]. It is reported that the medical cost of OP in China will increase to $581.97 billion by 2050, bringing a heavy burden to families and society [6]. Related studies of OP have shown that decreased activity of osteoblasts and enhanced differentiation of osteoclasts lead to a break in the balance between bone formation and bone resorption, which eventually leads to the onset of the disease [1,6]. At present, the main drugs used to treat OP are bone formation promoter, bone resorption inhibitor and bone mineralizer [7]. Bisphosphonates, estrogen receptor (selective) modulators and calcitonin are commonly used to relieve the corresponding symptoms. Although the effect is good, there are still some problems. For example, the drug holiday of bisphosphonates can only improve bone mineral density but not increase bone strength to reduce fracture risk. It has been reported that taking bisphosphonates for more than 5 years may increase the risk of atypical femoral fractures [7–9]. In addition, it has been reported that some of them may cause a range of non-negligible side effects, such as an increased risk of venous thromboembolism and esophageal cancer [7,9]. Estrogen and Selective Estrogen Receptor Modulators drugs have certain advantages in the treatment of postmenopausal osteoporosis, its main role is to reduce bone loss caused by bone resorption, and related studies have shown that it may lead to a decrease in the number of platelets [9]. Others, such as calcitonin and mineral supplements, treat primary op by inhibiting bone resorption without interfering with bone formation, and studies have reported that such drugs may cause limb pain, blood circulation disorders and severe stroke [10]. Since its approval more than 10 years ago, Denosumab has been a relatively good anti-osteoporosis targeted drug, and recently there have been reports of limb pain, stroke and other risks [11]. Therefore, we still need to find safer and more effective prevention strategies.

**Figure 1.**
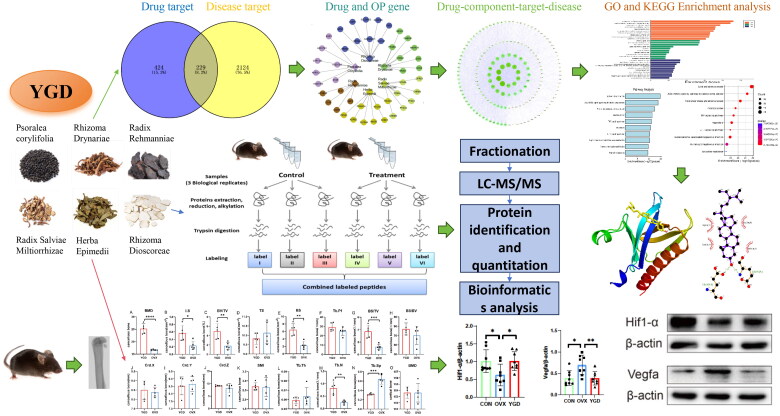
Research overview chart. The mechanism of action of YGD on OP was studied by network pharmacology, molecular docking, proteomics and *in vivo* animal experiments.

Yigu Decoction (YGD) is a clinical experience prescription for treating OP, which is composed of *Psoralea corylifolia*, *Rhizoma Drynariae*, *Herba Epimedii*, *Radix Rehmanniae*, *Salviae Miltiorrhizae* and *Rhizoma Dioscoreae*. Previous studies have shown that YGD has proved its therapeutic effect on OP to a certain extent in basic research [12,13] and clinical research [14,15]. Because of the complex components, multiple targets and wide levels of traditional Chinese medicine, the specific mechanism of its action on OP is not clear, and its components and key targets have not been determined. Based on the theory of systems biology, network pharmacology uses big data for network analysis, selects disease target genes and effective components of traditional Chinese medicine compound, and then designs multi-target drug molecules to systematically and comprehensively study the interaction of ‘drug-target-pathway-disease’. Its concept is actually consistent with the holistic view of TCM and the principle of syndrome differentiation and treatment. In this study, the active targets and substances of YGD were obtained by network pharmacological analysis, and preliminarily verified by molecular docking, followed by quantitative proteomics and animal experiments were used to verify its possible mechanism (Figure 1).

## 2. Materials and methods

### 2.1. Herbal preparation

YGD is provided by the Department of traditional Chinese Medicine Pharmacy, the Third Affiliated Hospital of Zhejiang Chinese Medical University, was identified as qualified medicinal materials. YGD combined of *Psoralea corylifolia 10 g*, *Rhizoma Drynariae 15 g*, *Herba Epimedii 15 g*, *Radix Rehmanniae* 15 g, *Radix Salviae Miltiorrhizae 30 g* and *Rhizoma Dioscoreae 15 g.* Soaking 30 min in the water of traditional Chinese medicine, then add 7 times the amount of water of the YGD-medicine, wait for the water to boil, start timing 30 min, and filter the liquid. The residue is then added with 5 times the amount of raw water, and after the water is boiled, the time 30 min begins and the liquid is filtered. After the twice extraction solution was mixed, it was concentrated by water bath heating, and finally concentrated to the water extract of 2 g/mL, which was stored in a low-temperature refrigerator. *Psoralea corylifolia (Cullen corylifolium (L.) Medik.), Rhizoma Drynariae (Drynaria roosii Nakaike), Herba Epimedii (Epimedium sagittatum (Siebold & Zucc.) Maxim.), Radix Rehmanniae (Rehmannia glutinosa (Gaertn.) DC.), Radix Salviae Miltiorrhizae (Salvia miltiorrhiza Bunge), Rhizoma Dioscoreae (Dioscorea oppositifolia L.).* The names of all drugs can be found on this website, http://mpns.kew.org. and the necessary checks were carried out on the web site.

Anti-HIF-1 alpha (ab179483), Anti-VEGFA (ab46154), Anti-Glucose Transporter GLUT1 (ab115730) and Anti-Osteoprotegerin (ab73400) were purchased from Abcam, UK. GAPDH Antibody – #AF7021 was purchased from Affinity Biosciences, China. Mouse Anti-β-Actin Monoclonal Antibody (DW9562) was purchased from DW, China.

### 2.2. The mechanism of YGD therapy for OP based on network pharmacology and molecular docking

Search for drug ingredients through the TCMSP platform (http://lsp, nwu.edu.cn/tcmsp. php), set oral availability (oral availability, OB) ≥ 30%, drug-like properties (drug- likeness, DL) ≥ 0.18. After obtaining the active ingredients, search for the action targets of single drug components through its MOL.ID number [16–17]. At the same time, the SMILES (Simplified Molecular Input Line Entry System) of the compound obtained by BATMAN and pubchem database (https://pubchem.ncbi.nlm.nih.gov) was imported into the structural similarity prediction target database Swiss Target Prediction (http://www.swisstargetprediction.ch) prediction target) [18–19]. The compound Excel data table was downloaded by Uniprot (http://www.uniprot.org/) database, the ‘TRIM’ function was used to optimize the data, and the ‘VLOOKU’ function was used to match the target gene name [20]. And supplement the unmatched gene names by consulting the literature. Finally, the target proteins of the chemical components obtained by the above methods are annotated using Uniprot database. The compound gene ‘network’ file and Type file were imported by Cytoscape 3.8.2 software, and the network topology analysis was carried out [21]. The target figure, color, transparency and size were adjusted according to the Degree value, and the ‘traditional Chinese medicine ingredient-target’ network diagram was constructed. Used GeneCards (https://www.genecards.org/), OMIM (https://www.omim.org/) and Drugbank (https://go.drugbank.com/) platforms to obtain disease-related targets, and the disease name takes ‘osteoporosis’ as the keyword to search osteoporosis disease-related targets. Set the object to ‘human’, use the ‘VLOOKUP’ function to match the target gene name, and screen the intersection genes of drug and disease [22]. The intersection targets of active compounds of traditional Chinese medicine and osteoporosis disease related targets were obtained by Venny (https://bioinfogp.cnb.csic.es/tools/venny/) software, which can be used as potential targets of traditional Chinese medicine compound in the treatment of osteoporosis. Through the String (https://string-db.org/) platform, the intersection gene was introduced, the object was set as (homo sapiens), the highest confidence level was 0.900, the free gene node was hidden, and the protein interaction was obtained. Results the network topology parameters were obtained by importing the Cytoscape3.7.2 software and selecting ‘network Analyzer’. The intermediate center value (Betweenness centrality, BC) is the number of times the shortest path passes through a single node, and the compact center value (closeness centrality, CC) is the degree of difficulty of communication between nodes. Take more than twice the median of Degree value and the median of BC and CC as the criteria to screen the core component targets of nourishing traditional Chinese medicine and diseases, make the PPI network interaction map [23].

In the R software (R3.6.0 software), the Bioconductor software package ‘org.Hs.eg.db’ is installed and run, and the common drug-disease target is converted into entrez ID. Then, install the ‘cluster Profiler’ package in R software, and perform functional enrichment analysis of key target genes Gene Ontology (GO) and KEGG (https://www.kegg.jp/) with *p* < 0.05 and *Q* < 0.05 according to the converted entrez ID [24], and output the results in the form of bar graph and bubble chart. The visual relationship among active ingredients, target proteins and signal pathways were established by using Cytoscape 3.8.0 software. After screening, the key genes of the effective components in the YGD for targeted intervention of osteoporosis were determined. These targets were confirmed by molecular docking. The crystal structures of the validation components are from the RCSB Protein Database (PDB, https://www.rcsb.Org) [25].

Molecular docking was performed using iGEM DOCK software. Download SDF format 3D structure from PubChem data according to CAS number of small molecules, import the structure into ChemBio3D Ultra 14.0 for energy minimization, set Minimum RMS Gradient to 0.001, and save small molecules in mol2 format. Import the optimized small molecule into AutodockTools-1.5.6 for hydrogenation, charge calculation, charge distribution, and set the rotable key, and save it as ‘pdbqt’ format. Download TNF (PDB ID: 2E7A), AKT1 (PDB ID: 1UNQ), EGFR (PDB ID: 2GS2), IL17 (PDB ID: 6WIO), RAGE (PDB ID: 4P2Y), using Pymol2.3.0 to remove the protein crystal water, the original ligand, etc., import the protein structure into AutoDocktools (v1.5.6) for hydrogenation, charge calculation, charge allocation, atom type designation and saving as ‘pdbqt’ format. POCASA 1.1 was used to predict protein binding sites, AutoDock Vina1.1.2 was used for docking, and TNF related parameters were set as: center_x = 12.7, center_y = 5.7, center_z = 13.7. Search space: size_x:50, size_y: 50, size_z:50 (spacing of each cell point is 0.375 A). Set AKT1 parameters to center_x = 14.7, center_y = 21.9, center_z = 16.6. Search space: size_x:50, size_y: 50, size_z:50 (spacing of each cell point is 0.375 A). EGFR parameters are set to center_x = 68.2, center_y = 18.6, and center_z = −40.3. Search space: size_x:50, size_y: 50, size_z:50 (spacing of each cell point is 0.375 A). IL17 Set related parameters to center_x = 67.5, center_y = 2.5, center_z =-8.73. Search space: size_x:50, size_y: 50, size_z:50 (spacing of each cell point is 0.375 A). RAGE parameters are set to: center_x = 17.1, center_y = 132.4, center_z = 39.8; Search space: size_x:50, size_y: 50, size_z:50 (spacing of each cell is 0.375 A), and all the remaining parameters are the default settings. All differentially expressed proteins were compared to all experimentally identified proteins with KEGG annotation results to reveal pathways of enrichment, as determined by Fisher’s exact test. *p* < 0.05 was considered statistically significant.

### 2.3. Experimental animal

In this study, 18 5-week-old female C57BL/6 mice, weighing 20 ± 3 g and pathogen-free, were used. All the experimental animals are provided by Shanghai Shrek Experimental Animal Co., Ltd. (license number: SCXK (Shanghai) 2017-0005). The experimental animals are raised by the Animal Experimental Center of Zhejiang Chinese Medical University (experimental facility license number: SYXK (Zhejiang) 2021-0012). The feeding room is well ventilated, the room temperature is controlled at 21 ± 1 °C, the humidity is 63%, the noise is less than 55 decibels, feed and drink at will, and alternate light/dark conditions every 12 h. All animals are given meticulous humanitarian care.18 mice were randomly divided into three groups: Control group (CON, *n* = 6), Ovariectomy group (OVX, *n* = 6) and YGD group (YGD, *n* = 6). After 1 week of adaptive feeding, the mice in the other two groups except CON group were given intraperitoneal injection of ketamine 5 mg/100g and then bilateral ovariectomy was performed. 3 days after operation, each group of ovariectomized mice was injected with 40,000 units of penicillin per day to prevent infection, and no mice died after operation. After operation, the mice in each group were routinely fed for 8 weeks, and from the 9th week, the mice in CON group and OVX group were fed with 0.2 mL/20g double distilled water, and the mice in YGD group were given water extract from 0.2 mL/20g YGD at 9: 00 am every day, once a day for 8 weeks. After intragastric administration, 10% pentobarbital sodium was anesthetized by intraperitoneal injection, the left ventricle was intubated with normal saline, and the bilateral femurs and tibia were quickly frozen and stored in the refrigerator at −80 °C.

### 2.4. Determination of BMD in mice

The bone mineral density (BMD) of 5 mice in each group was randomly measured by DXA (InAlyzer), and the bone microstructure of right femur was measured by Micro-CT (Bruker Sky scan 1176).

### 2.5. Extraction, classification and identification of proteins

The high-abundance protein was removed from the serum of mice, and the protein was extracted by the cleavage method of SDT (4%(w/v) SDS, 100 mM Tris/HCl pH7.6, 0.1 M DTT), and then the protein was quantified by BCA method. Appropriate amount of protein was obtained from each sample for tryptic enzymatic hydrolysis using Filter aided proteome preparation (FASP) method, and the peptide was desalt using C18 Cartridge. The peptide was lyophilized and redissolved with 40 μL 0.1% formic acid solution. Quantitative peptide (OD280). 100 μg peptide segments were taken from each sample and labeled according to Thermo’s TMT labeling kit instructions. LC-MS/MS analysis was carried out on the Q Exactive mass spectrometer (Thermo Fisher Scientific), which was coupled with Easy nLC (Proseon Biosystems, now Thermo Fisher Scientific) for 90 min. The MASCOT engine embedded in Proteome Discoverer 1.4 (Matrix Science, London, UK; version 2.2) was used to search the MS raw data of each sample for identification and quantitative analysis [26,27].

### 2.6. Bioinformatics analysis

The quantitative information of the target protein set was normalized (normalized to the interval (−1,1)). The Complexheatmap R package (R Version 3.4) was used to simultaneously classify both the sample and protein expression dimensions (distance algorithm: Euclidean, linkage method: Average linkage) and generate hierarchical clustering heat maps. The process of GO annotation of a target protein collection using Blast2GO can be roughly summarized into four steps: sequence comparison (Blast), GO entry extraction (Mapping), GO annotation (Annotation), and Inter Pro Scan supplemental annotation (Annotation Augmentation). The KAAS (KEGG Automatic Annotation Server) software was utilized to perform KEGG pathway annotation on the target protein collection. Enrichment analysis of GO annotation or KEGG pathway annotation was performed on the target protein collection by comparing the distribution of each GO classification or KEGG pathway in the target protein collection and the overall protein collection using Fisher’s Exact Test (FET). The information in the STRING (http://string-db.org/) database was used to find the interaction relationships between the target proteins, and the interaction networks were generated and analyzed using CytoScape software [28,29].

### 2.7. Western blotting

The left femur tissue of each group of mice was taken. Fully grind the bone tissue in liquid nitrogen, grind the powdered bone tissue into the 1.5 mL EP tube, add RIPA lysate and protease inhibitor without EDTA, add 2 grinding beads and put into the grinder to grind the bone tissue fully. BCA protein detection kit (plygen, China, B1511) was used to quantify the total protein. The same amount of protein was separated by sodium dodecyl sulfate polyacrylamide gel electrophoresis (SDS-PAGE) and then transferred to polyvinylidene fluoride (PVDF) membrane. After blocking, the first antibody was incubated overnight at 4 C. The first antibodies used were mouse β-actin polyclone (1: 5000) and rabbit Gapdh (1:7000), rabbit HIF-1 alpha (1:1000), rabbit VEGFA (1: 1000), rabbit Glucose Transporter GLUT1(1:100,000). TBST was washed and then goat anti-rabbit and mouse immunoglobulin G were incubated with horseradish peroxidase for 2 h at room temperature. The protein bands were displayed by enhanced chemiluminescence (enhanced chemuminescence, ECL) substrate, and the gray values were analyzed by ImageJ software.

### 2.8. Statistical analysis

All data comes from at least three independent experiments, expressed as mean ± standard deviation (*x* ± *s*). Analyze univariate comparisons between two groups using Student’s *T*-test in GraphPad Prism software, and compare multivariate data through analysis of variance. The difference was statistically significant (*p* < 0.05).

## 3. Results

### 3.1. Screening of active components and corresponding target proteins of YGD in treating OP

**Figure 2.**
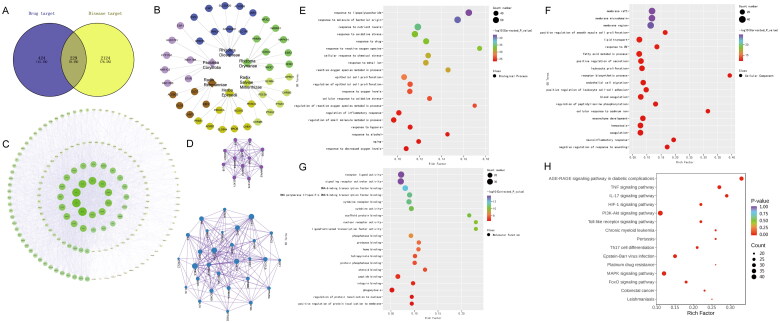
Active components, targets, component interactions and bioinformatics analysis of YGD therapy for OP based on network pharmacology. (A) The Wayne diagram of eight points related to drugs and diseases. (B) The target map of the active components of the drug. (C) Drug-component-target-disease network. (D) Protein interaction network. (E–H) KEGG enrichment analysis and GO enrichment analysis.

A total of 167 effective active ingredients were obtained, and 424 drug targets were screened out by inputting the targets corresponding to the active ingredients into UniProt database and using Swiss Target Prediction database. The obtained compounds and targets are used to construct pharmacological networks. With ‘OP’ as the key word, they were searched in GeneCards database, and 2124 disease targets were obtained after de-duplication. On the Venny2.1 online software mapping tool platform, 424 drug targets and 2124 disease targets were input, and veen diagram was drawn. After the intersection of the two, 229 common drug-disease targets were obtained (Figure 2(A,B), Table 1), More detailed documentation can be found in Supplementary Images 1.

**Table 1.** Basic information statistics table of TCM-components-targets of the drug group.

Chinese herb	Number of components	Number of predicted targets
Radix Salviae Miltiorrhizae	68	138
Rhizoma Drynariae	20	174
Rhizoma Dioscoreae	16	59
Herba Epimedii	25	225
Radix Rehmanniae	9	104
Psoralea corylifolia	29	336

### 3.2. Drug-component-target-disease network

Through the String platform, we imported the intersecting genes, set the object as (homo sapiens), took the highest confidence level of 0.900, and hid the free gene nodes to get the protein interactions. The network diagram of ‘drug-ingredient-target-disease’ interaction was drawn (Figure 2(C)). The analysis of the network diagram by using Network Analyzer shows that the most central positions are tumor necrosis factor (TNF), vascular endothelial growth factor (VEGFA), mitogen-activated protein kinase 3 (MAPK3), serine-threonine protein kinase B (AKT), epidermal growth factor receptor (EGFR), Insulin (INS), interleukin 6 (IL-6), Interleukin 1β (IL-1β), and so on.

### 3.3. Protein interaction network

The above-mentioned 424 common targets were recorded in the STRING database, and the PPI network and potential targets of protein interaction were obtained by analysis (Figure 2(D)). Through the connection lines of the PPI network, we can see that HIF-1α, EGFR, VEGFA, AKT1, MAPK3, TNF, IL6, ESR1 and other proteins are more important proteins in the interaction network.

### 3.4. KEGG enrichment analysis and GO enrichment analysis

In order to explain more specifically the potential mechanism of the influence of the active ingredients of YGD on OP, the biological process, cellular components and molecular functions of 424 common targets were selected by GO analysis after running in R language (Figure 2(E–G)). GO results showed that there were 3148 biological processes in the intersection gene set, including steroid reaction, nutrient metabolism, steroid metabolism, gland development, oxidative emergency response, aging, cell hypoxia response, energy metabolism, cell apoptosis, cell proliferation and so on. The expression of 123 cell components mainly involves cell membrane region, membrane transport, neuronal cell body, synaptic contact, cell matrix connection and so on; 224 of them bind to molecular functions, including receptor ligands, DNA and RNA transcription, nuclear receptors, transcription factors, steroids and so on. After running 434 common targets in R language, 175 KEGG pathways were obtained. The top 20 results formed a bar graph of KEGG function enrichment, and P represented the significance of enrichment. The redder the color, the higher the significance (Figure 2(H)). The results showed that the common targets were mainly enriched in AGE-RAGE signaling pathway, TNF signaling pathway, IL-17 signaling pathway, HIF-1 signaling pathway, PI3K-AKT signaling pathway, etc. By reviewing published studies, above all of the 5 signaling pathways may be involved to some extent in the development of OP. The above pathway may play a role in promoting bone formation and inhibiting bone resorption, as well as regulating osteogenic differentiation of bone marrow mesenchymal stem cells and inhibiting their lipogenesis. In addition, there are reports indicating that it may be involved in regulating energy metabolism and other pathways to improve bone metabolism.

### 3.5. Molecular docking results analysis

**Figure 3.**
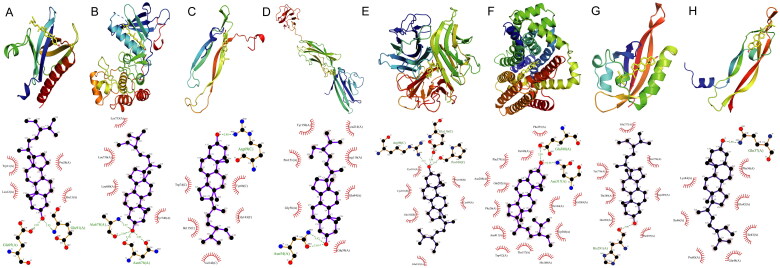
The results of molecular docking. (A) Stigmasterol with AKT1, (B) Stigmasterol with EGFR, (C) flavonoids with IL-17, (D) Stigmasterol with RAGE, (E) Stigmasterol with TNF, (F) flavonoids with HIF-1α, (G) Stigmasterol with VEGFA, (H) Stigmasterol with GLUT1.

The core of the Stigmasterol was considered YGD compounds. According to the result of KEGG pathway enrichment analysis, we chose the interaction between neural activity and receptor signaling pathways, choose their genetic crossing with the OP different biological functions and the largest proportion of genes, signal channel using PyMOL2.3.0 and Ligplot V2.2.5 interaction mode analysis was carried out on the docking results. Based on the corresponding relationship between drugs and the target, by molecular lock target protein pathways. Using the Auto dock Vina software, molecular docking can value the lowest five target protein and active ingredient Stigmasterol connection (AKT1, EGFR, IL-17, RAGE, TNF, HIF-1α, VEGFA, GLUT1). The combination of Stigmasterol and AKT1 for −7.3 kcal/mol, EGFR for −8.3 kcal/mol, IL-17 for −7.8 kcal/mol, RAGE for −6.6 kcal/mol, TNF for −9.3 kcal/mol, HIF-1α for −7.4 kcal/mol, VEGFA for −6.8 kcal/mol, GLUT1 for −10.3 kcal/mol (Figure 3(A–H)). The results show that the above YGD active ingredient is through the combined with target and give play to the role of treatment.

### 3.6. Improvement of bone morphology in OP mice by YGD

**Figure 4.**
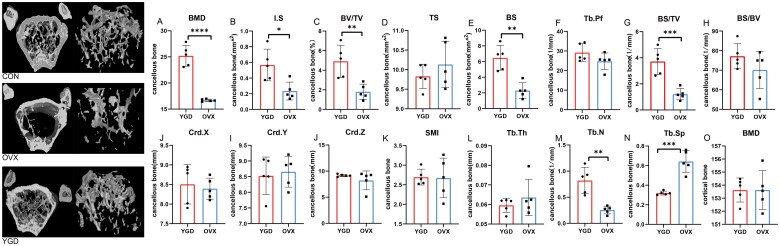
The results of bone microstructure of mice treated with Micro-CT. Figure 3. A-N represents the comparison of cancellous bone between the two groups of mice, and Figure 3. O represents the comparison of the bone cortex of the two groups of mice. *For *p* value < 0.05, ** for *p* value < 0.01, *** for *p* value < 0.005, **** for *p* < 0.001.

The bone mineral density of five ovariectomized mice was measured randomly, which showed that the model of ovariectomized mice was successful (*p* < 0.01), and the bone mineral density was significantly improved after intragastric administration of YGD for 8 weeks (*p* < 0.01), see as Table 2. The data processed by Micro-CT showed that YGD had obvious advantages in improving BMD, intersecting surface (I.S.), Bone surface (BS), Bone surface density (BS/TV), Trabecular number (Tb.n), Trabecular separationbone (Tb.sp) in ovariectomized mice (Figure 4). It shows that YGD has a certain ability to improve the bone tissue of OP mice.

**Table 2.** Comparison of bone density among different groups of rats (g/cm^2^, *x* ±* s*).

GROUP	N	BMD
CON	5	0.222 ± 0.007
OVX	5	0.162 ± 0.005**
YGD	5	0.204 ± 0.005^ΔΔ^

*Note.* Compared to the normal group,

** *p* < 0.01; Compared with the model group,

ΔΔ *p* < 0.01.

### 3.7. Effect of YGD intervention on serum protein expression in OP mice

**Figure 5.**
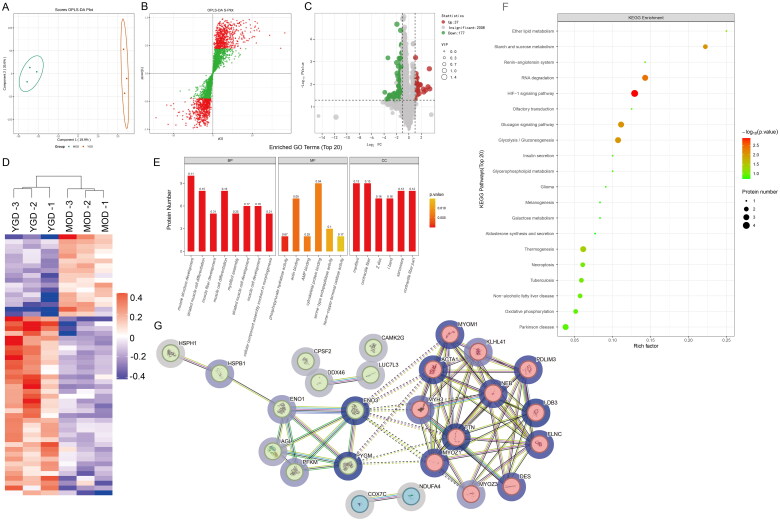
TMT quantitative proteomics revealed the possible mechanism of YGD affecting OP. (A) Principal component analysis based on OPLS-DA. (B) Differential product analysis based on OPLS-DA. (C) The volcanic map of the difference between YGD group and CON group. (D) Cluster analysis based on differential products. (E–G) Bioinformatics analysis based on YGD and CON group.

TMT quantitative proteomic analysis was used to further explore the mechanism of YGD intervention on OP. Firstly, the principal components of the two groups were analyzed by orthogonal partial least square discriminant analysis (OPLS-DA), and the substances that were different between the two groups were analyzed by OPLS-DA S-plot (Figure 5(A,B)). In order to analyze the differentially expressed proteins between the two groups, the differential metabolic proteins were screened by volcanic map, and a total of 214 proteins were significantly affected after treatment. Compared with the model group, 37 proteins were up-regulated and 177 down-regulated in YGD group. To demonstrate the significant differences of proteins between the comparison groups, the proteins in the comparison groups were plotted in a volcano plot using the two factors of expression difference fold change (Fold change) and *p* value (*T*-test) as the criteria, in which the proteins that were significantly down-regulated were marked in green color (FC < 0.833 and *p* < 0.05), and the proteins that were significantly up-regulated were marked in red color (FC > 1.2 and *p* < 0.05), and proteins with no difference are in gray, as follows (Figure 5(C)). Next, the significance of the influence of the change of differential protein expression on the sample was further determined, and the differential proteins in the comparison group were grouped by hierarchical clustering algorithm and presented in the form of heat map. With the screening criteria of fold change >1.2-fold and *p* value <0.05 (*T*-test or otherwise), the significantly differentially expressed proteins obtained can effectively separate the comparison groups (Figure 5(D)). We carried out functional annotation by GO and KEGG analysis to further explain the biological function of differential protein expression after YGD treatment. The functional categories in which all differentially expressed proteins were enriched were found by comparing all differentially expressed proteins against the annotation results of all proteins of the reference species in terms of GO/KEGG function, and the significance of the differences was derived by FET (*p* value < 0.05). Bubble plots were used to show the enrichment of GO entries under each of the three major GO categories (Figure 5(E,F)). Among the GO items, 1054 biological processes (BP) were mainly detected muscle structure development, striated muscle cell differentiation, et al. 239 cellular components (CC) were mainly expressed myofibril, contractile fiber, *Z* disc, et al. 214 molecular functions (MF) were mainly reflected phosphor pyruvate hydratase activity and actin binding, et al. (Figure 5(E)). By consulting the literature, it is found that the biological processes with OP conduit include muscle structure development, striated muscle cell differentiation, contractile fiber, actin binding, et al. In order to analyze the biological process and the mechanism of drug action more systematically, we analyzed the KEGG pathway enrichment of 214 differential proteins. The signal pathways related to OP mainly include HIF-1 signaling pathway, Glucagon signaling pathway, Glycolysis/Gluconeogenesis, Starch and sucrose metabolism, et al. (Figure 5(F)). According to PPI network analysis, 23 proteins can interact directly among the 214 differentially expressed proteins, which is YGD method for proteins to participate in the biological process of the body. Referring to the literature, it is found that Ttn, Ldb3, Pygm, Eno3, Acta1 and other genes are involved in the biological occurrence and development of OP.

### 3.8. Western blotting (WB) to verify the expression of key proteins in HIF signaling pathway

**Figure 6.**
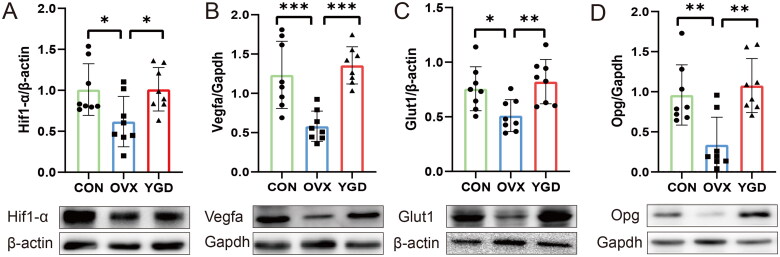
WB to verify the expression of key proteins in HIF signaling pathway. *For *p* value < 0.05, ** for *p* value < 0.01, *** for *p* value < 0.005.

Based on the network pharmacology and proteomics enrichment analysis of YGD, we found that the HIF signal pathway was significantly enriched. Hypoxia inducible factor (HIF) is a transcription factor that responds to changes in available oxygen in the cellular environment, especially to oxygen depletion or hypoxia. HIF-1 α regulates glucose transporter (Glut1), glycolytic enzyme, vascular endothelial growth factor (VEGF) and other gene protein products to increase oxygen transport or promote hypoxic metabolism. Based on this, we used WB to detect the expression of related proteins in HIF signal pathway after YGD intervention, in order to explain the possible mechanism of traditional Chinese medicine YGD in the treatment of OP. In order to further determine the possible mechanism of YGD interfering with OP, we also used WB to determine the expression of bone formation-related proteins to support the possible role of YGD. The results showed that in HIF signal pathway, the expression of HIF1- α protein in OP model decreased, but increased significantly after YGD intervention (*p* < 0.05). The downstream protein VEGFA was obviously expressed in OP model, and the expression of VEGFA was inhibited by YGD intervention (*p* < 0.01). On the contrary, the expression of Glut1 protein decreased in OP model, and it was promoted by YGD intervention (*p* < 0.01). It has reported that Osteoprotegerin (OPG) plays a key role in bone remodeling and has a protective effect on bone. The expression of OPG in OP mice was significantly increased after intervention with YGD (*p* < 0.01) (Figure 6).

## 4. Discussion

OP, as a systemic metabolic bone disease, is characterized by low bone mass and destruction of bone tissue microstructure. Its onset is hidden, its incidence is increasing year by year, and its fracture risk is high [6,28–32], resulting in huge social burden [29,31]. At present, it is considered that the pathogenesis of OP is related to osteoblasts and osteoclasts [1,8,9], mainly because the balance of bone reconstruction is broken, the imbalance between bone absorption and bone formation, and the osteogenesis ability is low, and the osteoclast ability is greater than the osteogenesis ability. It has been proved that YGD can play a key role in bone metabolism by regulating many osteogenic functional pathways such as classic PI3K/AKT, Wnt and BMP pathway [12–15,33,34]. Because of the complex composition of Chinese patent medicine compound, the mechanism of its influence on OP is still unclear. The potential targets and related mechanisms of YGD in the treatment of OP were determined by means of network pharmacology, proteomics and experimental verification.

Network pharmacology research has found that the traditional Chinese medicine compound YGD contains effective ingredients such as stigmasterol, flavonoids, quercetin, luteolin, kaempferol and psoralen. Currently, research on the relationship between these ingredients and OP has been reported. Stigmasterol is a kind of plant sterol, which can lower cholesterol and has anti-inflammatory and immunomodulatory effects [35,36]. Some related studies have confirmed that stigmasterol has advantages in improving OP. Through the study of network pharmacology and molecular docking, Lu et al. found that stigmasterol is an important target and active component involved in the OP process [37]. Qu et al. verified that stigmasterol can improve the bone mineral density of osteoporotic rats, which may be achieved by improving the expression of related proteins and genes [38]. Quercetin is a kind of natural flavonoids, which is extracted from many plants. Sina et al. intervened ovariectomized rats by quercetin, which showed that OP caused by ovariectomy could be alleviated by regulating autophagy and apoptosis of rat bone cells [37,39]. Kaempferol shows its bi-directional regulation in the treatment of OP from the aspects of protecting bone formation and inhibiting bone resorption. It can protect osteoblasts and reduce the functional expression of osteoclasts through anti-inflammation, inhibition of oxidative stress, autophagy and other mechanisms, so as to achieve the purpose of treatment [40–42]. Related studies on psoralen have shown that psoralen can improve the level of estrogen after ovarian aging, thus reducing the ability of bone resorption to prevent and treat OP [43,44]. In a previous study, higher levels of soy sterols were found in YGD as determined by UV-visible spectroscopy. In this study, stigmasterol, the effective component of YGD, has a strong binding correlation with AKT1, EGFR, IL-17, RAGE, TNF, HIF-1 α, VEGFA and GLUT1, indicating that the improvement of OP by YGD may be achieved through many aspects. In earlier studies, we have demonstrated that YGD can improve OP by inhibiting osteoclast proliferation and improving bone resorption through PI3K/AKT pathway [13,45]. Earlier studies on the promotion of osteoblast proliferation by YGD have shown that it can promote the expression of osteoblast protein and function through WNT/BMP-2 classical signal pathway so as to achieve the purpose of prevention and treatment of OP [46].

On the basis of the established drug-component-target-disease network diagram, we can easily see that the key factors are protein kinases AKT, MAPK3, IL-6, TNF, ESR1 and ESR2, insulin closely related to glucose metabolism, and growth factors VEGF and EGFR. Obviously, these targets are related to bone metabolism to a certain extent, which will be the potential target proteins of YGD affecting bone parameters. Protein kinases AKT and MAPK are the central nodes of many signal pathways, which can mediate many biological functions, such as cell proliferation, survival, glucose metabolism, protein synthesis and genome stability [47,48]. Interestingly, MAPK was originally discovered as an enzyme protein activated by insulin [49]. Insulin can combine with insulin receptor on osteoblast surface, so as to stimulate the accumulation of amino acids in osteoblasts and promote the formation of collagen [50]. TNF indirectly activates mature osteoclasts and inhibits the apoptosis of osteoclasts by acting on osteoblasts. TNF is an important inducer of other factors that regulate the differentiation of osteoclast precursor cells into mature osteoclasts, such as IL-6, M-CSF and GM-CSF [51]. The role of IL-6 in OP mainly acts on the early stage of osteoclasts, showing its influence on osteoclasts and their precursors, which can not only affect their production quantity, but also affect their functions [36,52]. ESR is the main mediator of biological effects of estrogen and selective estrogen receptor modulators [53]. As far as the bone system is concerned, ESR1 can be expressed in osteoblasts, osteoclasts, chondrocytes, bone marrow stromal cells and bone cells. estrogen can change related enzyme activities such as MAPK by targeting estrogen receptors in chondrocytes [54]. At present, VEGF is one of the most clearly studied angiogenic factors. In recent years, it has been found that as a bone growth factor, VEGF directly interferes with bone formation and reconstruction and plays an important role in bone metabolism [55]. EGFR signal can maintain the number of superficial chondrocytes, promote the secretion of boundary lubricant and lubrication of cartilage surface, and enhance the mechanical strength of articular cartilage [56]. PI3K signaling is activated downstream of growth factor receptors, including PDGF receptor (PDGFR) and EGFR, which drives cell proliferation and migration [57]. It can be seen that there is a tandem reaction between the target proteins, and this association effect makes a slight change in the level of target proteins cause a great change in bone metabolism. Based on the results of this study, we should study the influence of TCM on specific targets at molecular level in the future to find the most critical target protein.

In addition, YGD not only acts on the key signaling pathways of OP such as PI3K-AKt signaling pathway, estrogen receptor signaling pathway, Wnt signaling pathway, osteoclast differentiation. Our study has also proved that insulin signal pathway, AGE-RAGE signal pathway, HIF-1 signal pathway and IL-17 signal pathway at the same time, and works synergistically on multiple sites and key pathways. the study suggests that YGD has advantages of multi-level, multi-target and multi-link in anti-osteoporosis, and dynamically regulates organism pathology. The corresponding 3148 genes/target proteins were found by network pharmacology method, which showed that YGD components played a certain role in all aspects of OP such as cell function activation and metabolic function regulation to energy metabolism, apoptosis, autophagy, etc. and had a dual regulation effect on osteoclast differentiation and osteoblast proliferation, and had a regulation effect on other systems related to bone metabolism. At the same time, it showed the rationality and effectiveness of YGD components in invigorating kidney and spleen, strengthening muscles and bones, promoting blood circulation and relieving pain, etc. Based on TMT quantitative proteomics, it was found that HIF1 signaling pathway was significantly enriched, which was consistent with the prediction of network pharmacology.

Most of the studies on HIF signaling pathway are related to tumors and cardiovascular diseases. HIF signaling pathway plays a role in two cases, under normoxia state and hypoxia state. Under normoxia condition, hif1- α will be hydroxylated and play a further role. Under hypoxia, hif-1 α is not hydroxylated to stabilize the protein and enhance transcriptional activation [58]. At present, related studies on OP believe that aging and hypoxia are the key mechanisms of OP production, and the study of HIF1 signal pathway may be a feasible way to prevent and further treat OP. It has reported that some studies have revealed the relationship between HIF1 signal pathway and OP. The results show that HIF regulating the expression of downstream related proteins VEGF and GLUT1 is the possible mechanism that affects the occurrence and development of OP [59,60]. The current research on the relationship between HIF- α and OP is mainly through the study of its regulation of osteoblast and osteoclast function, but its specific mechanism is still unclear. Some studies suggest that hypoxia leads to the activation of downstream VEGF expression by HIF1- α and promotes the proliferation and differentiation of osteoclasts [61,62]. On the contrary, a considerable number of studies suggest that the high expression of HIF1- α under hypoxia plays a role in protecting osteoblasts [63–66]. In this study, the results of WB verification after the intervention of YGD in ovariectomized mice showed that the high expression of HIF1- α may also indicate that the high expression of HIF under hypoxia may protect the function of osteoblasts and promote the ability of bone formation. It has been reported that GLUT1 is closely related to bone formation. As one of the main glucose transporters, ATP is mainly released by glycolysis to induce osteoblast differentiation [67–69]. Under the action of HIF1- α, it promotes the high expression of GLUT1, suggesting that YGD may affect downstream energy metabolism through this pathway, thus promoting the function of osteoblasts [68,69]. VEGFA has been recognized by many researchers for its excellent functions in bone formation, bone protection and injury repair [67,69–74]. Like most studies, our results also confirmed that HIF1- α can activate the expression of downstream VEGFA and promote its osteogenic function. It also shows that YGD may activate downstream VEGFA and GLUT1 simultaneously by mediating HIF1- α. Because the expression of functions such as bone formation and bone protection required energy, the activation of GLUT1 provides a functional pathway for VEGFA to perform its function. OPG can inhibit the apoptosis of endothelial cells and promote the maturation of vascular endothelial cells. At the same time, OPG and RANKL can also inhibit the differentiation of osteoclasts [75]. The result of YGD intervention in OPG confirmed the function of VEGFA from the side. On the other hand, it also shows that YGD may not only promote osteogenic function, but also inhibit the expression of osteoclasts.

In modern medicine, anti-osteoporosis emphasizes a single target, and the population is limited, so the side effects and side effects of drugs are inevitable. We should pay attention to the development and application of multi-objective, multi-level and multi-link drug anti-OP, which has greater social significance and economic value to the broad audience. However, it is difficult to clarify the specific mechanism of multi-component, multi-channel and multi-target therapy of traditional Chinese medicine compound prescription such as YGD on OP. By constructing a more complex drug-target-disease network analysis method, network pharmacology changes the traditional pharmacology research of traditional Chinese medicine from a single target to a comprehensive network analysis, which accords with the research reality. By predicting the action mechanism of various components in the compound prescription of traditional Chinese medicine, using reasonable bioinformatics methods and experimental verification has important application value for in-depth study of the anti-osteoporosis mechanism of natural plant drugs.

In spite of this, there are still some shortcomings in this study, that is, only part of the experiment *in vivo* has been done, and there is no experimental verification of molecular docking and binding of all the active components of drugs to related proteins. At the same time, the verification of HIF signal pathway needs to be further deepened to fully reveal the more complete mechanism of action of YGD on OP.

## 5. Conclusion

In short, we used network pharmacology and molecular docking technology to explore the potential drug targets and molecular mechanisms of YGD in the treatment of OP, and preliminarily verified by molecular docking. Further through animal experiments, after the intervention of YGD in OP mice, histomorphological analysis was carried out to verify the function of YGD, and the possible mechanism of YGD was preliminarily verified by TMT quantitative proteomics. Finally, the HIF signal pathway enriched by KEGG was found from network pharmacology and proteomics, and the related proteins were verified by WB. It was confirmed that YGD may achieve the purpose of treating OP by promoting the proliferation of osteoblasts and protecting the function of osteoblasts through hif1- α/vegf/glut1 pathway. The research results provide a promising direction for further research in the future, which is of great significance to reveal the exact regulation mechanism.

## Supplementary Material

Supplemental Material

## Data Availability

Data from the study are provided in supplementary materials. Some proteomic data may be obtained upon reasonable request by email corresponding author XM, Yao at yxmzcmu@163.com.

## References

[1] Anderson PA, Freedman BA, Brox WT, et al. Osteoporosis: recent recommendations and positions of the American Society for Bone and Mineral Research and the International Society for Clinical Densitometry. J Bone Joint Surg Am. 2021;103(8):741–747. doi:10.2106/JBJS.20.01248. 33587517

[2] Sun J, Yao C, Wang Z, et al. The beneficial effects of square dance on musculoskeletal system in early postmenopausal Chinese women: a cross-sectional study. BMC Womens Health. 2022;22(1):247. doi:10.1186/s12905-022-01832-9. 35729521 PMC9215099

[3] Zhao J-G, Zeng X-T, Wang J, et al. Association between calcium or vitamin D supplementation and fracture incidence in community-dwelling older adults: a systematic review and meta-analysis. JAMA. 2017;318(24):2466–2482. doi:10.1001/jama.2017.19344. 29279934 PMC5820727

[4] Gao Q, Hu K, Yan C, et al. Associated factors of sarcopenia in community-dwelling older adults: a systematic review and meta-analysis. Nutrients. 2021;13(12):4291. 27 Nov. doi:10.3390/nu13124291. 34959843 PMC8707132

[5] Li N, Zheng B, Liu M, et al. Cost-effectiveness of antiosteoporosis strategies for postmenopausal women with osteoporosis in China. Menopause. 2019;26(8):906–914. doi:10.1097/GME.0000000000001339. 30994577

[6] Si L, Winzenberg TM, Jiang Q, et al. Projection of osteoporosis-related fractures and costs in China: 2010-2050. Osteoporos Int. 2015;26(7):1929–1937. doi:10.1007/s00198-015-3093-2. 25761729

[7] Watts NB, Camacho PM, Lewiecki EM, et al. American Association of Clinical Endocrinologists/American College of Endocrinology clinical practice guidelines for the diagnosis and treatment of postmenopausal osteoporosis-2020 update. Endocr Pract. 2021;27(4):379–380. doi:10.1016/j.eprac.2021.02.001. 33577971

[8] Sánchez A, Blanco R. Osteonecrosis of the jaw (ONJ) and atypical femoral fracture (AFF) in an osteoporotic patient chronically treated with bisphosphonates. Osteoporos Int. 2017;28(3):1145–1147. doi:10.1007/s00198-016-3840-z. 27866217

[9] Moshi MR, Nicolopoulos K, Stringer D, et al. The Clinical effectiveness of denosumab (prolia^®^) for the treatment of osteoporosis in postmenopausal women, compared to bisphosphonates, Selective Estrogen Receptor Modulators (SERM), and Placebo: a systematic review and network meta-analysis. Calcif Tissue Int. 2023;112(6):631–646. doi:10.1007/s00223-023-01078-z. 37016189

[10] Eastman K, Gerlach M, Piec I, et al. Effectiveness of parathyroid hormone (PTH) analogues on fracture healing: a meta-analysis. Osteoporos Int. 2021;32(8):1531–1546. doi:10.1007/s00198-021-05847-0. 33559713

[11] Kendler DL, Cosman F, Stad RK, et al. Denosumab in the treatment of osteoporosis: 10 years later: a narrative review. Adv Ther. 2022;39(1):58–74. doi:10.1007/s12325-021-01936-y. 34762286 PMC8799550

[12] Zhang R, Yan K, Wu Y, et al. Quantitative proteomics reveals the effect of Yigu decoction (YGD) on protein expression in bone tissue. Clin Proteomics. 2021;18(1):24. doi:10.1186/s12014-021-09330-0. 34641785 PMC8513338

[13] Li N, Gong Y. The mechanism of the Yigutang-mediated P13K/AKT/GSK-3β signal pathway to regulate osteogenic differentiation of bone marrow stromal stem cells to treat osteoporosis. Evid Based Complement Alternat Med. 2021;2021:6699781. doi:10.1155/2021/6699781. 34239593 PMC8233090

[14] Zhang R-K, Yan K, Chen H-F, et al. Anti-osteoporotic drugs affect the pathogenesis of gut microbiota and its metabolites: a clinical study. Front Cell Infect Microbiol. 2023;13:1091083. doi:10.3389/fcimb.2023.1091083. 37475958 PMC10354646

[15] Chen Z, Xie L, Xu J, et al. Changes in alkaline phosphatase, calcium, C-reactive protein, D-dimer, phosphorus and hemoglobin in elderly osteoporotic hip fracture patients. Ann Palliat Med. 2021;10(2):1079–1088. doi:10.21037/apm-20-218. 33040555

[16] Ru J, Li P, Wang J, et al. TCMSP: a database of systems pharmacology for drug discovery from herbal medicines. J Cheminform. 2014;6(1):13. doi:10.1186/1758-2946-6-13. 24735618 PMC4001360

[17] Huang L, Xie D, Yu Y, et al. TCMID 2.0: a comprehensive resource for TCM. Nucleic Acids Res. 2018;46(D1):D1117–D1120. doi:10.1093/nar/gkx1028. 29106634 PMC5753259

[18] Liu Z, Guo F, Wang Y, et al. BATMAN-TCM: a bioinformatics analysis tool for molecular mechANism of traditional Chinese medicine. Sci Rep. 2016;6(1):21146. doi:10.1038/srep21146. 26879404 PMC4754750

[19] Gfeller D, Grosdidier A, Wirth M, et al. Swiss Target Prediction: a web server for target prediction of bioactive small molecules. Nucleic Acids Res. 2014;42(Web Server issue):W32–W38. doi:10.1093/nar/gku293. 24792161 PMC4086140

[20] UniProt Consortium T. UniProt: the universal protein knowledgebase. Nucleic Acids Res. 2018;46(5):2699. doi:10.1093/nar/gky092. 29425356 PMC5861450

[21] Doncheva NT, Morris JH, Holze H, et al. Cytoscape stringApp 2.0: analysis and visualization of heterogeneous biological networks. J Proteome Res. 2023;22(2):637–646. doi:10.1021/acs.jproteome.2c00651. 36512705 PMC9904289

[22] Fishilevich S, Nudel R, Rappaport N, et al. GeneHancer: genome-wide integration of enhancers and target genes in GeneCards. Database (Oxford). 2017;2017:bax028. doi:10.1093/database/bax028. 28605766 PMC5467550

[23] Szklarczyk D, Morris JH, Cook H, et al. The STRING database in 2017: quality-controlled protein-protein association networks, made broadly accessible. Nucleic Acids Res. 2017;45(D1):D362–D368. doi:10.1093/nar/gkw937. 27924014 PMC5210637

[24] Chen L, Chu C, Lu J, et al. Gene ontology and KEGG pathway enrichment analysis of a drug target-based classification system. PLoS One. 2015;10(5):e0126492. doi:10.1371/journal.pone.0126492. 25951454 PMC4423955

[25] Bhat TN, Bourne P, Feng Z, et al. The PDB data uniformity project. Nucleic Acids Res. 2001;29(1):214–218. doi:10.1093/nar/29.1.214. 11125095 PMC29799

[26] Wiśniewski JR, Zougman A, Nagaraj N, et al. Universal sample preparation method for proteome analysis. Nat Methods. 2009;6(5):359–362. doi:10.1038/nmeth.1322. 19377485

[27] Zhou Q, Xie F, Zhou B, et al. Differentially expressed proteins identified by TMT proteomics analysis in bone marrow microenvironment of osteoporotic patients. Osteoporos Int. 2019;30(5):1089–1098. doi:10.1007/s00198-019-04884-0. 30739146

[28] Xiao J, Zhang G, Mai J, et al. Bioinformatics analysis combined with experimental validation to explore the mechanism of XianLing GuBao capsule against osteoarthritis. J Ethnopharmacol. 2022;294:115292. doi:10.1016/j.jep.2022.115292. 35447200

[29] Aibar-Almazán A, Voltes-Martínez A, Castellote-Caballero Y, et al. Current status of the diagnosis and management of osteoporosis. Int J Mol Sci. 2022;23(16):9465. doi:10.3390/ijms23169465. 36012730 PMC9408932

[30] Cauley JA. Osteoporosis: fracture epidemiology update 2016. Curr Opin Rheumatol. 2017;29(2):150–156. doi:10.1097/BOR.0000000000000365. 28072591

[31] Borgström F, Karlsson L, Ortsäter G, et al. Fragility fractures in Europe: burden, management and opportunities. Arch Osteoporos. 2020;15(1):59. doi:10.1007/s11657-020-0706-y. 32306163 PMC7166207

[32] Schoeneberg C. Current management of hip fracture. Medicina (Kaunas). 2022;59(1):26. doi:10.3390/medicina59010026. 36676650 PMC9862304

[33] Li G, Yao X. Experimental study of Yigu Decoction on the expression of osteoblast-related genes in osteoporotic rats. J Zhejiang Chin Med Uni. 2018;10(53):727–729.

[34] Lin X, Fang F, Peng Z, et al. Effect of Yigu Decoction on Wnt/β-catenin classic signaling pathway in bone tissue of ovariectomized rats. J Zhejiang Chin Med Uni. 2018;42(02)97–104.

[35] Ahmad Khan M, Sarwar AHMG, Rahat R, et al. Stigmasterol protects rats from collagen induced arthritis by inhibiting proinflammatory cytokines. Int Immuno­pharmacol. 2020;85:106642. doi:10.1016/j.intimp.2020.106642. 32470883

[36] Sampath SJP, Rath SN, Kotikalapudi N, et al. Beneficial effects of secretome derived from mesenchymal stem cells with stigmasterol to negate IL-1β-induced inflammation in-vitro using rat chondrocytes-OA management. Inflammopharmacology. 2021;29(6):1701–1717. doi:10.1007/s10787-021-00874-z. 34546477

[37] Lu Z, Huang M, Lin H, et al. Network pharmacology and molecular docking approach to elucidate the mechanisms of Liuwei Dihuang pill in diabetic osteoporosis. J Orthop Surg Res. 2022;17(1):314. doi:10.1186/s13018-022-03194-2. 35701780 PMC9195436

[38] Ou L, Kang W, Liang Z, et al. Investigation of anti-osteoporosis mechanisms of Rehmanniae Radix Preparata based on network pharmacology and experimental verification. J Orthop Surg Res. 2021;16(1):599. doi:10.1186/s13018-021-02751-5. 34649566 PMC8515747

[39] Martiniakova M, Babikova M, Mondockova V, et al. The role of macronutrients, micronutrients and flavonoid polyphenols in the prevention and treatment of osteoporosis. Nutrients. 2022;14(3):523. doi:10.3390/nu14030523. 35276879 PMC8839902

[40] Wong SK, Chin K-Y, Ima-Nirwana S, et al. The osteoprotective effects of kaempferol: the evidence from *in vivo* and *in vitro* studies. Drug Des Devel Ther. 2019;13:3497–3514. doi:10.2147/DDDT.S227738. PMC6789172 31631974

[41] Sharma AR, Nam J-S. Kaempferol stimulates WNT/β-catenin signaling pathway to induce differentiation of osteoblasts. J Nutr Biochem. 2019;74:108228. doi:10.1016/j.jnutbio.2019.108228. 31678747

[42] Gan L, Leng Y, Min J, et al. Kaempferol promotes the osteogenesis in rBMSCs via mediation of SOX2/miR-124-3p/PI3K/Akt/mTOR axis. Eur J Pharmacol. 2022;927:174954. doi:10.1016/j.ejphar.2022.174954. 35421359

[43] Lu Y, Zhang M, Zhang J, et al. Psoralen prevents the inactivation of estradiol and treats osteoporosis via covalently targeting HSD17B2. J Ethnopharmacol. 2023;311:116426. doi:10.1016/j.jep.2023.116426. 36997132

[44] Li H, Deng W, Qin Q, et al. Isoimperatorin attenuates bone loss by inhibiting the binding of RANKL to RANK. Biochem Pharmacol. 2023;211:115502. doi:10.1016/j.bcp.2023.115502. 36921635

[45] He B, Zhu Y, Ying J, et al. Yigu Tang containing medicated serum promoting the proliferation and differentiation of osteoblast through classic Wnt signaling pathway. New Traditional Chinese Medicine. 2017;49(03):10–13.

[46] Yan K, Chen H, Zhang R, et al. Eficacy of Yigu decoction on the signal pathway of nuclear factor kb receptor activator ligand in ovariectomized osteoporosis rats. Chin J Trad Med. 2022;30(09):1–6.

[47] Vakili S, Zal F, Mostafavi-Pour Z, et al. Quercetin and vitamin E alleviate ovariectomy-induced osteoporosis by modulating autophagy and apoptosis in rat bone cells. J Cell Physiol. 2021;236(5):3495–3509. doi:10.1002/jcp.30087. 33030247

[48] Casado-Díaz A, Anter J, Dorado G, et al. Effects of quercetin, a natural phenolic compound, in the differentiation of human mesenchymal stem cells (MSC) into adipocytes and osteoblasts. J Nutr Biochem. 2016;32:151–162. doi:10.1016/j.jnutbio.2016.03.005. 27142748

[49] Prouillet C, Mazière J-C, Mazière C, et al. Stimulatory effect of naturally occurring flavonols quercetin and kaempferol on alkaline phosphatase activity in MG-63 human osteoblasts through ERK and estrogen receptor pathway. Biochem Pharmacol. 2004;67(7):1307–1313. doi:10.1016/j.bcp.2003.11.009. 15013846

[50] Licini C, Vitale-Brovarone C, Mattioli-Belmonte M, et al. Collagen and non-collagenous proteins molecular crosstalk in the pathophysiology of osteoporosis. Cytokine Growth Factor Rev. 2019;49:59–69. doi:10.1016/j.cytogfr.2019.09.001. 31543432

[51] Lavrador P, Gaspar VM, Mano JF, et al. Bioinspired bone therapies using naringin: applications and advances. Drug Discov Today. 2018;23(6):1293–1304. doi:10.1016/j.drudis.2018.05.012. 29747006 PMC7617200

[52] Dong G-C, Ma T-Y, Li C-H, et al. A study of *Drynaria fortunei* in modulation of BMP–2 signalling by bone tissue engineering. Turk J Med Sci. 2020;50(5):1444–1453. doi:10.3906/sag-2001-148. 32252500 PMC7491309

[53] Shishido K, Reinders A, Asuthkar S, et al. Epigenetic regulation of radioresistance: insights from preclinical and clinical studies. Expert Opin Investig Drugs. 2022;31(12):1359–1375. doi:10.1080/13543784.2022.2158810. 36524403

[54] Brägelmann J, Lorenz C, Borchmann S, et al. MAPK-pathway inhibition mediates inflammatory reprogramming and sensitizes tumors to targeted activation of innate immunity sensor RIG-I. Nat Commun. 2021;12(1):5505. doi:10.1038/s41467-021-25728-8. 34535668 PMC8448826

[55] Sturgill TW, Ray LB. Muscle proteins related to microtubule associated protein-2 are substrates for an insulin-stimulatable kinase. Biochem Biophys Res Commun. 1986;134(2):565–571. doi:10.1016/s0006-291x(86)80457-0. 3511906

[56] Berman NK, Honig S, Cronstein BN, et al. The effects of caffeine on bone mineral density and fracture risk. Osteoporos Int. 2022;33(6):1235–1241. doi:10.1007/s00198-021-05972-w. 34981132

[57] Wang T, He C. TNF-α and IL-6: the link between immune and bone system. Curr Drug Targets. 2020;21(3):213–227. doi:10.2174/1389450120666190821161259. 31433756

[58] Gonzalez FJ, Xie C, Jiang C, et al. The role of hypoxia-inducible factors in metabolic diseases. Nat Rev Endocrinol. 2018;15(1):21–32. doi:10.1038/s41574-018-0096-z. 30275460 PMC6624429

[59] Shao J, Liu S, Zhang M, et al. A dual role of HIF1α in regulating osteogenesis-angiogenesis coupling. Stem Cell Res Ther. 2022;13(1):59. doi:10.1186/s13287-022-02742-1. 35123567 PMC8818171

[60] Bie M, Tang Y, Xia Y, et al. HIF-1α mediates osteoclast-induced disuse osteoporosis via cytoophidia in the femur of mice. Bone. 2023;168:116648. doi:10.1016/j.bone.2022.116648. 36563716

[61] Wang J, Zhang Y, Cao J, et al. The role of autophagy in bone metabolism and clinical significance. Autophagy. 2023;19(9):2409–2427. doi:10.1080/15548627.2023.2186112. 36858962 PMC10392742

[62] Song X, Tang Y, Zhu J, et al. HIF-1α induces hypoxic apoptosis of MLO-Y4 osteocytes via JNK/caspase-3 pathway and the apoptotic-osteocyte-mediated osteoclastogenesis in vitro. Tissue Cell. 2020;67:101402. doi:10.1016/j.tice.2020.101402. 32835935

[63] Zhang D, Du J, Yu M, et al. Urine-derived stem cells-extracellular vesicles ameliorate diabetic osteoporosis through HDAC4/HIF-1α/VEGFA axis by delivering microRNA-26a-5p. Cell Biol Toxicol. 2023;39(5):2243–2257. doi:10.1007/s10565-022-09713-5. 35554780

[64] Li L, Li A, Zhu L, et al. Roxadustat promotes osteoblast differentiation and prevents estrogen deficiency-induced bone loss by stabilizing HIF-1α and activating the Wnt/β-catenin signaling pathway. J Orthop Surg Res. 2022;17(1):286. doi:10.1186/s13018-022-03162-w. 35597989 PMC9124388

[65] Xu K, Lu C, Ren X, et al. Overexpression of HIF-1α enhances the protective effect of mitophagy on steroid-induced osteocytes apoptosis. Environ Toxicol. 2021;36(11):2123–2137. doi:10.1002/tox.23327. 34310007

[66] Yin N, Zhu L, Ding L, et al. MiR-135-5p promotes osteoblast differentiation by targeting HIF1AN in MC3T3-E1 cells. Cell Mol Biol Lett. 2019;24(1):51. doi:10.1186/s11658-019-0177-6. 31410089 PMC6686269

[67] Zhao Q, Shen X, Zhang W, et al. Mice with increased angiogenesis and osteogenesis due to conditional activation of HIF pathway in osteoblasts are protected from ovariectomy induced bone loss. Bone. 2012;50(3):763–770. doi:10.1016/j.bone.2011.12.003. 22193550

[68] Lee W-C, Guntur AR, Long F, et al. Energy metabolism of the osteoblast: implications for osteoporosis. Endocr Rev. 2017;38(3):255–266. doi:10.1210/er.2017-00064. 28472361 PMC5460680

[69] Zoidis E, Ghirlanda-Keller C, Schmid C, et al. Stimulation of glucose transport in osteoblastic cells by parathyroid hormone and insulin-like growth factor I. Mol Cell Biochem. 2011;348(1–2):33–42. doi:10.1007/s11010-010-0634-z. 21076856

[70] Yang J, Li S, Li Z, et al. Targeting YAP1-regulated glycolysis in fibroblast-like synoviocytes impairs macrophage infiltration to ameliorate diabetic osteoarthritis progression. Adv Sci (Weinh). 2024;11(5):e2304617. doi:10.1002/advs.202304617. 38044289 PMC10837355

[71] Yu F, Chang J, Li J, et al. Protective effects of oridonin against osteoporosis by regulating immunity and activating the Wnt3a/β-catenin/VEGF pathway in ovariectomized mice. Int Immunopharmacol. 2023;118:110011. doi:10.1016/j.intimp.2023.110011. 36924567

[72] Liu Y, Berendsen AD, Jia S, et al. Intracellular VEGF regulates the balance between osteoblast and adipocyte differentiation. J Clin Invest. 2012;122(9):3101–3113. doi:10.1172/JCI61209. 22886301 PMC3428080

[73] Regan JN, Lim J, Shi Y, et al. Up-regulation of glycolytic metabolism is required for HIF1α-driven bone formation. Proc Natl Acad Sci U S A. 2014;111(23):8673–8678. doi:10.1073/pnas.1324290111. 24912186 PMC4060724

[74] Xu Z, Wang P, Wang Z, et al. ER-β accelerates the process of primary osteoporosis by promoting VEGFA-mediated apoptosis of osteoblasts. Genomics. 2023;115(6):110743. doi:10.1016/j.ygeno.2023.110743. 37967683

[75] Li Y, Yang S, Yang S, et al. Rb1 negatively regulates bone formation and remodeling through inhibiting transcriptional regulation of YAP in Glut1 and OPG expression and glucose metabolism in male mice. Mol Metab. 2022;66:101630. doi:10.1016/j.molmet.2022.101630. 36343919 PMC9672361

