# Supplementary figures and images for: Using network pharmacology and molecular docking technology, proteomics and experiments were used to verify the effect of Yigu decoction (YGD) on the expression of key genes in osteoporotic mice
Source: Ann Med. 2025 Jan 3;57(1):2449225. doi: 10.1080/07853890.2024.2449225 (PMC11703118; doi:10.1080/07853890.2024.2449225)

Figure.S1 Compounds target network for core YGD components.


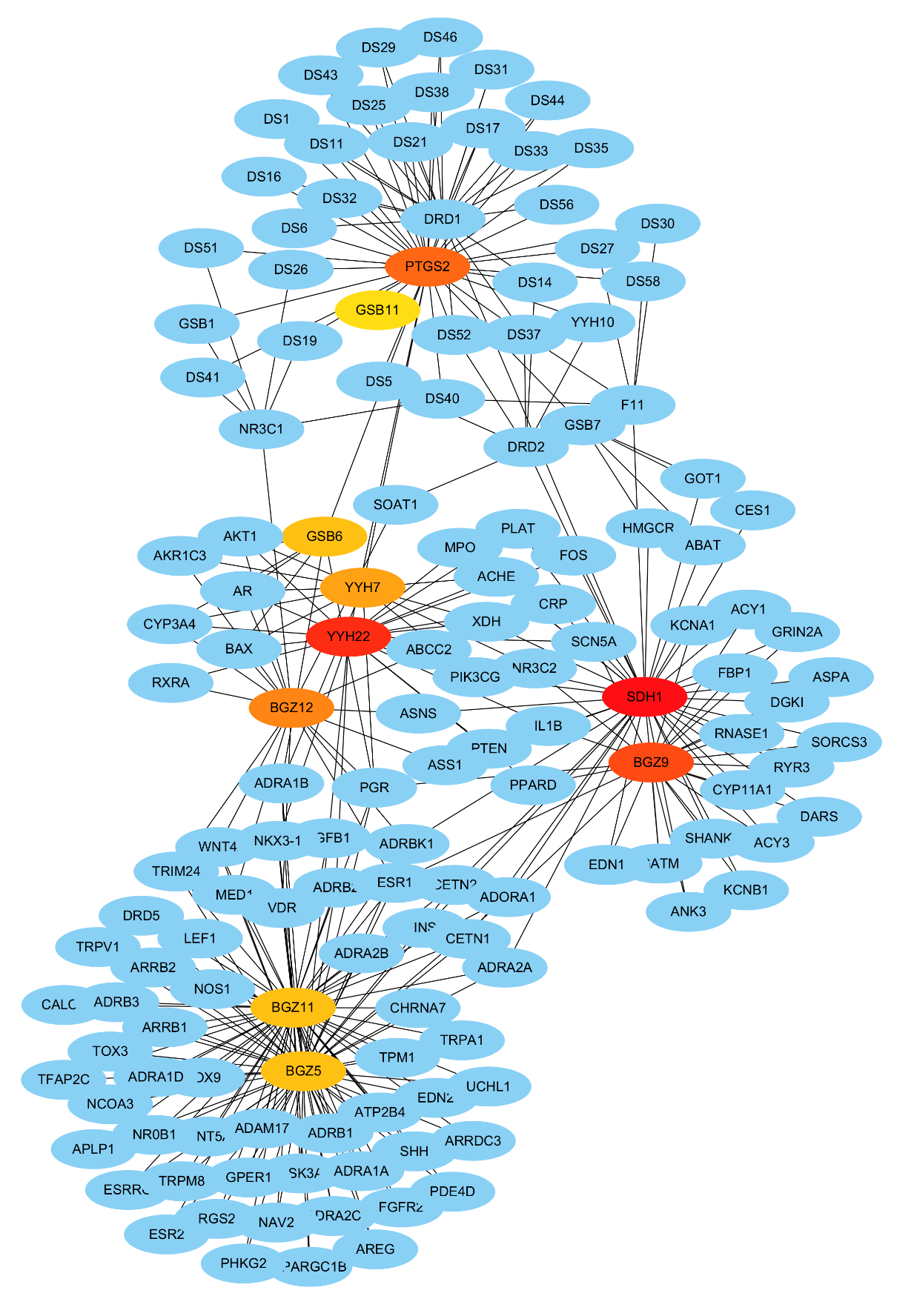

Supplement: Supplemental Material [file IANN_A_2449225_SM6634.zip › Suppl_Mat/Suppl_Fig.docx]
